# DyGAT-FTNet: A Dynamic Graph Attention Network for Multi-Sensor Fault Diagnosis and Time–Frequency Data Fusion

**DOI:** 10.3390/s25030810

**Published:** 2025-01-29

**Authors:** Hongjun Duan, Guorong Chen, Yuan Yu, Chonglin Du, Zhang Bao, Denglong Ma

**Affiliations:** 1 School of Intelligent Technology and Engineering, Chongqing University of Science and Technology, No. 20 Daxuecheng East Road, Shapingba District, Chongqing 401331, China2022208004@cqust.edu.cn (C.D.); 2024208019@cqust.edu.cn (Z.B.); 2 XJTU School of Mechanical Engineering, Xi’an Jiaotong University, Xi’an 710049, China; denglong.ma@xjtu.edu.cn

**Keywords:** multi-sensor system, fault diagnosis, graph neural network, dynamic graph structure

## Abstract

Fault diagnosis in modern industrial and information systems is critical for ensuring equipment reliability and operational safety, but traditional methods have difficulty in effectively capturing spatiotemporal dependencies and fault-sensitive features in multi-sensor data, especially rarely considering dynamic features between multi-sensor data. To address these challenges, this study proposes DyGAT-FTNet, a novel graph neural network model tailored to multi-sensor fault detection. The model dynamically constructs association graphs through a learnable dynamic graph construction mechanism, enabling automatic adjacency matrix generation based on time–frequency features derived from the short-time Fourier transform (STFT). Additionally, the dynamic graph attention network (DyGAT) enhances the extraction of spatiotemporal dependencies by dynamically assigning node weights. The time–frequency graph pooling layer further aggregates time–frequency information and optimizes feature representation.Experimental evaluations on two benchmark multi-sensor fault detection datasets, the XJTUSuprgear dataset and SEU dataset, show that DyGAT-FTNet significantly outperformed existing methods in classification accuracy, with accuracies of 1.0000 and 0.9995, respectively, highlighting its potential for practical applications.

## 1. Introduction

In modern industrial and information technology systems, multi-sensor systems are widely used for equipment condition monitoring and diagnostics to ensure stable system operation and prevent potential failures. Among these, fault detection is a critical component, aiming to accurately identify abnormal behaviors which significantly deviate from normal operating conditions. With the advancement of sensor technology, multi-sensor systems can provide rich data, offering possibilities for in-depth analysis and understanding of system states. However, efficiently extracting useful information from these multi-source data and constructing accurate fault detection models remains a significant challenge. In recent years, advancements in artificial intelligence (AI) have significantly enhanced the field of fault diagnosis, particularly in the integration of multi-sensor data [1,2]. Multi-sensor data fusion integrates outputs from multiple sensors, capturing comprehensive and diverse information while addressing the limitations of incomplete single-sensor data [3]. However, research on integrating and fusing multi-source data remains insufficient.Thus, there is an urgent need to advance this field by developing more robust fault diagnosis models to enhance predictive analysis and offer deeper insights into system operations.

Traditional fault detection methods often rely on manually setting static thresholds to analyze the time series signals of each sensor. However, this approach often falls short when dealing with large-scale data, and its effectiveness is limited by the quality of feature extraction [4,5]. To address this issue, deep learning technology has been widely applied in intelligent fault diagnosis in recent years due to its strong automatic feature extraction capabilities [6,7]. For instance, Wang et al. [8] proposed a multi-task convolutional neural network (CNN) which avoids the influence of local minima by automatically extracting generalized information; Wen et al. [9] transformed time-domain fault signals into RGB image formats and used a pretrained ResNet-50 model to extract features for fault diagnosis; and Chen et al. [10] designed a long short-term memory (LSTM) network combined with physical information for more accurate fault prediction. In addition, time–frequency analysis methods (such as the short-time Fourier transform and wavelet transform) are also used to capture the non-stationary characteristics of signals, providing richer feature representations for fault diagnosis [11,12,13]. For example, Zhao et al. [14] proposed CTNet, a data-driven time–frequency analysis (TFA) technique combining a fully convolutional autoencoder network and a convolutional block attention module (CBAM) to address the challenge of characterizing non-stationary fault signals with closely spaced or crossed instantaneous frequencies. Ding et al. [15] proposed a novel time–frequency transformer (TFT) model. The TFT leverages the success of the Transformer architecture in sequence processing and introduces customized tokenizer and encoder modules. This end-to-end framework performs well on the bearing experiment dataset. However, these methods mostly focus on processing single-sensor signals, neglecting the complex spatial dependencies (such as topological structures and modal correlations) and temporal dependencies (such as cycles and trends) between multi-sensor signals, leading to insufficiencies in the processing of multi-source information [16].

To address the issue of handling multi-source information in fault diagnosis, the application of information fusion methods in fault diagnosis has gradually gained attention, especially in the field of multi-sensor data fusion. Fault diagnosis methods based on information fusion can fully explore spatiotemporal structural information by combining signals from different sensors, thereby enhancing diagnostic accuracy [17]. Shao et al. [18] proposed a semi-supervised learning method for intelligent fault diagnosis of offshore wind turbines, which realizes feature fusion of vibration signals and acoustic emission signals through coupled convolutional residual networks, improving diagnostic accuracy. Wen et al. [19] proposed a hybrid CNN-Wiener model in this study, which effectively enhances the deep learning prediction performance of the remaining useful life (RUL) through feature-level multi-sensor fusion and the construction of health indices. Zhang et al. [20] proposed a multi-modal data cross-domain fusion network which uses vibration signals and thermal images to comprehensively capture the health status of gearboxes. Ye et al. [21] proposed a multi-sensor residual convolutional fusion network (MRCFN) for intelligent fault diagnosis of bearings, addressing challenges such as strong noise interference and insufficient fault samples. The method integrates a convolutional pooling module (CPM) with a double ring residual module (DRRM) for rough and deep feature extraction from multi-sensor signals, avoiding network performance degradation. A spatial channel reconstruction module (SCRM) is introduced to eliminate redundant information and enhance training efficiency. Finally, a global interactive perception fusion module (GIPFM) and classification block (CB) are used to globally fuse features from acoustic and vibration signals, enabling high-precision fault identification. Xiao et al. [22] proposed a multi-level information fusion fault diagnosis method for rotating machinery, utilizing multilayer graph data, attention-based feature fusion, and information entropy-based decision fusion to enhance diagnostic accuracy and robustness. In addition, Li et al. [23] designed an enhanced selective ensemble deep learning method based on the beetle antenna search (BAS) algorithm and introduced an enhanced weighted voting (EWV) combination strategy with class-specific thresholds to improve diagnostic accuracy rates. Although fusion methods at different levels have shown broad potential in fault diagnosis, they also face challenges such as data redundancy, high feature dimensionality, and decision ambiguity, which require further optimization.

In mechanical equipment fault detection, it is crucial to deploy various heterogeneous sensors. These sensors are distributed in different locations of the equipment and can capture anomalies in the spatial dimension of the system as well as periodic fault characteristics in the temporal dimension [24]. In order to further analyze multi-sensor signal data, graph neural networks (GNNs) based on graph structures have been widely used. Using methods such as graph convolutional networks (GCNs) and Chebyshev spectral convolutional networks (ChebyNet), the computational complexity is effectively reduced, and efficient implementation of graph convolution operations is achieved [25]. On this basis, GNNs have been successfully used to model spatiotemporal dependencies and achieved remarkable results in traffic prediction, mechanical fault diagnosis, and other fields [26]. For example, Xu et al. [27] proposed a graph-guided collaborative convolutional neural network (GGCN) for highly effective fault diagnosis of electromechanical systems, which explores inherent correlations between multi-source signals through a graph reasoning fusion module (GRFM) and considers both the distribution gaps and intrinsic correlations among signals simultaneously, improving diagnostic accuracy, particularly under noisy conditions. Wang et al. [28] proposed a novel spatiotemporal graph neural network with an attention-aware module (A-TSGNN) for intelligent fault diagnosis. The network uses graph structure to organize multi-sensor signals and adopts graph convolution and temporal learning to achieve spatiotemporal feature representation. Compared with single-sensor schemes and other fusion models, it shows an excellent diagnostic ability and stable performance. Shao et al. [29] proposed a multi-scale cluster graph convolution neural network with a multi-channel residual network (MR-MCGCN) for machine fault diagnosis, which addresses the challenges of extracting weak features under variable load conditions and the unknown receptive scale of GCNs. The method combines a multi-channel residual network (MCRN) for weak feature extraction, an autoencoder (AE) graph generation layer for creating finite graph data at different scales, and a multi-scale cluster graph convolution neural network for intelligent fault diagnosis. The experimental results for three datasets demonstrate that the MR-MCGCN achieved superior diagnostic accuracy compared with the other methods, even under variable load conditions. However, existing methods mainly focus on spatial convolution and insufficiently mine the time–frequency information and dynamic dependencies in multi-sensor data, which limits the comprehensive performance of fault detection. Therefore, how to extract key features from signals while capturing the complex relationships between multiple sensors has become an important research direction to improve the accuracy of fault detection.

GNNs have demonstrated significant potential in extracting fault-sensitive feature representations from multi-sensor data. This paper introduces a fault diagnosis model leveraging multi-sensor graph neural networks. Sensors are represented as nodes, and their outputs are segmented for processing. A learnable dynamic graph construction (LDGC) method is used to generate a dynamic adjacency graph. The generated graph is subsequently input into a dynamic graph attention network, which learns the relative importance of sensors and their connections. After being processed by the dynamic graph attention network, the data area passed through a time–frequency graph pooling layer to enhance feature learning. Finally, a fully connected layer recognizes the fault states.

The main contributions of this study are summarized as follows:

(1) A new learnable dynamic graph construction method is proposed for dynamically constructing time–frequency graphs. This method combines learnable embedding vectors with distance metrics to automatically generate adjacent matrices based on the similarity or distance between nodes. The adjacency matrix is continuously optimized during training, enhancing the adaptability and expressive power of the graph structure in feature learning. This approach effectively captures time–frequency dependencies and improves the model’s performance in sequential data processing.

(2) A dynamic graph attention network (DyGAT) is proposed to effectively handle and learn the spatiotemporal dependencies between different time slots. Using the dynamic graph transformation (DGT) method, the graph data of the current time slot are combined with the graph data from the previous time slot, establishing temporal relationships. By introducing a graph attention mechanism, the DyGAT dynamically assigns different weights to each node, focusing on more important parts of the graph and thereby enhancing the model’s ability to learn temporal features.

(3) A time–frequency graph pooling layer is introduced to enhance the model’s feature learning ability on time–frequency data. This pooling layer extracts local features from the time–frequency graph through convolution operations and incorporates adaptive weight parameters with the adjacency matrix in a graph convolution mechanism to dynamically adjust feature representations. Through adaptive learning and time–frequency dependency modeling, the time–frequency graph pooling layer can more effectively aggregate time–frequency information, thus improving the model’s performance on complex time–frequency data.

The structure of this paper is organized as follows. Section 2 reviews the related work. Section 3 details the DyGAT-FTNet model. Section 4 validates the proposed method using two representative datasets from different experimental platforms. Finally, Section 5 concludes the paper.

## 2. Related Work

This section provides a concise overview of the related work, focusing on time–frequency signal fault detection and GNNs.

### 2.1. Time-Frequency Signal Fault Detection

Time–frequency signals play a crucial role in equipment fault detection. Traditional methods, such as the STFT and wavelet transform (WT), are widely used in rotating machinery fault detection by converting signals from the time domain to the time–frequency domain, effectively extracting features related to frequency variations over time [30,31,32,33]. However, the fixed window size in the STFT limits the flexibility of the time and frequency resolution, while the WT is sensitive to noise at different scales and struggles to adapt to non-stationary signals under complex operating conditions. With the increasing demand for analyzing nonlinear and non-stationary signals, modern methods such as empirical mode decomposition (EMD) and variational mode decomposition (VMD) have gained attention [34,35,36]. These methods can adaptively decompose signals and extract intrinsic features from different modes, making them more suitable for processing complex mechanical fault signals [37,38]. However, when handling high-dimensional and multi-modal signals, these methods are prone to issues like decomposition instability and feature redundancy, which reduce the efficiency and accuracy of fault feature extraction.

In recent years, the combination of time–frequency analysis and deep learning has become a research hotspot in the fault detection field [39]. By using time–frequency spectrograms as input, CNNs [40] or LSTM [41] are employed to extract time–frequency features, enabling efficient and accurate fault classification. Wang et al. [8] proposed the multi-task convolutional neural network (PKT-MCNN), which constructs a knowledge structure from coarse to fine, effectively addressing the issue of poor convergence in large-scale fault diagnosis caused by class-internal/class-external distance imbalances in feature space. At the same time, Xu et al. [42] developed an unsupervised learning method which combines stacked denoising autoencoders (SDAEs) and Gath–Geva (GG) clustering for diagnosing rolling bearing faults under different operating conditions. Liang et al. [43] utilized the wavelet transform and an improved residual neural network for feature extraction, enhancing robustness against label noise. Cheng et al. [44] combined the continuous wavelet transform with local binary convolutional layers to achieve faster training speeds and improved prediction accuracy. Wang et al. [45] proposed an automated, ultrasonic-based diagnosis approach for evaluating compressive damage in concrete amidst temperature variations, integrating continuous wavelet transforms and transfer learning-enhanced deep convolutional neural networks. This method addresses the vulnerability of ultrasonic-based non-destructive testing to environmental temperature changes by conducting tests on pre-damaged concrete specimens under varying temperature and damage conditions. The results demonstrate that the proposed approach effectively identifies concrete damage states despite temperature fluctuations and suggests that the training set’s temperature range should uniformly cover the expected lifespan temperature range of concrete structures. This study provides new insights for ultrasonic testing of concrete under environmental variations. These methods outperform traditional approaches in terms of feature learning capability but still face challenges in multi-modal data fusion and real-time performance.

Most existing methods focus on extracting time–frequency features from single-sensor data, neglecting the correlation and dynamic variations between different sensor data. This study aims to develop a time–frequency fusion-based graph neural network model, which improves fault detection accuracy under complex operating conditions by fusing the time–frequency domain features of different sensor data.

### 2.2. Graph Neural Network

In complex industrial systems, sensors often have highly intricate topological dependencies and information exchanges, which traditional fault detection methods struggle to effectively capture. To address this challenge, GNNs have emerged as an important tool in the field of fault detection due to their powerful modeling capabilities for non-Euclidean data [46]. Among the many applications of deep learning, the attention mechanism has proven to be an efficient and widely applicable method for data processing [47]. Graph attention networks (GATs) combine GNNs with the attention mechanism, determining the weight of information transmission between nodes through learnable attention coefficients and breaking through the limitations of fixed weights in traditional GCNs, thereby enhancing the expressive power of graphs and achieving significant results in tasks such as traffic flow prediction and time series modeling [28,48].

In a GNN, the representation of a node is typically aggregated from the information of its neighboring nodes. In a standard GCN, the new representation of a node *v* is represented by

(1) $$h_{v}^{\left(k+1\right)}=\sigma \left(\sum_{u\in N(v)}W^{\left(k\right)}h_{u}^{\left(k\right)}\right),$$

where $h_{v}^{\left(k+1\right)}$ is the feature representation of node *v* at layer *k*, $N_{u}$ is the set of neighbors of node $v_{o}$, *W* is the weight matrix at layer *k*, and σ is a nonlinear activation function.

In GATs, information transfer between nodes is governed by learnable attention coefficients rather than fixed-adjacency matrix weights. The weights between adjacent nodes are computed using a self-attention mechanism. The self-attention mechanism in a GAT determines the importance of a neighboring node’s features to a central node. This process involves first transforming the node features with a shared weight matrix *W*, followed by calculating the attention coefficients $e_{vu}$ for each pair of nodes *v* and *u* using a parameter vector ${\vec{a}}^{T}$.

The formula for computing the attention coefficients is

(2) $$e_{vu}=\mathrm{LeakyReLU}\left({\vec{a}}^{T}\left[W{\vec{h}}_{v}∥W{\vec{h}}_{u}\right]\right),$$

where $e_{uv}$ represents the attention value from node *u* to node *v*, *W* represents learnable parameters, and ‖ denotes the concatenation operation.

After calculating the initial attention scores, these scores are normalized for each node using the softmax function to obtain the final attention coefficients $\alpha_{vu}$:

(3) $$\alpha_{vu}=\frac{\exp(e_{vu})}{\sum_{k\in N_{v}}\exp\left(e_{vu}\right)},$$

where $\alpha_{vu}$ represents the normalized attention coefficients and $N_{v}$ is the set of neighbors of node *v*.

The GAT can be expressed as follows:

(4) $$h_{v}^{\left(k+1\right)}=\sigma \left(\sum_{u\in N(v)}\alpha_{vu}Wh_{u}^{\left(k\right)}\right)$$

Fault detection methods based on GCNs and GATs have been widely applied in various scenarios [49]. Li et al. [50] introduced a multi-receptive field graph convolutional network (MRF-GCN) for efficient fault diagnosis by integrating features from multiple receptive fields. Zhou et al. [51] developed a method based on dynamic graphs and few-shot feature learning, constructing new graph representations for noisy samples to enhance diagnostic performance under varying signal-to-noise ratios.

Despite the strong performance of GNNs in fault detection, current methods exhibit several shortcomings. First, existing methods often assign equal weights to data from different sensors, neglecting their varying responses to different faults. Second, the dynamic topological dependencies between sensors over time have not been fully explored.

To address these limitations, this study proposes DyGAT-FTNet, a multi-sensor fault detection method based on dynamic graph attention mechanism networks. By generating multi-sensor graphs using learnable dynamic graph construction and DyGAT, the proposed method significantly improves fault classification accuracy.

## 3. Method

In this section, we will introduce the proposed DyGAT-FTNet. Section 3.1 will introduce the overall framework of the model. The remaining sections will introduce each component in the model.

### 3.1. Overview of Model Architecture

**Figure 1:**
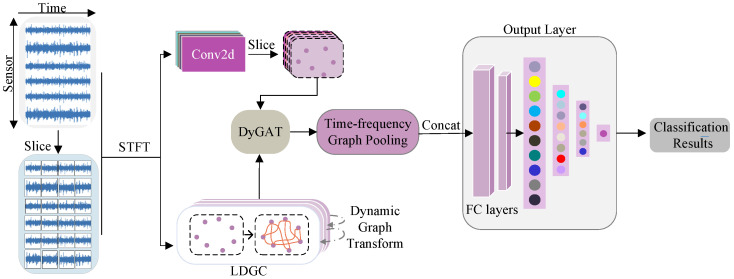
DyGAT-FTNet processes multi-sensor signals by segmenting them into time slots and extracting time–frequency information via an STFT. Dynamic multi-sensor graphs are constructed and processed using graph attention and DyGAT. The time–frequency graph pooling layer optimizes features, and the output module performs fault classification using pooling and fully connected layers.

We begin by briefly introducing the overall framework of the proposed DyGAT-FTNet model. As shown in Figure 1, the DyGAT-FTNet model consists of several key components. Initially, the multi-sensor signals are segmented into multiple time slots, with the length of each slot determined by a sliding window. To construct the graph-based relationships between different sensors, we transform the data within each time slot into time–frequency information through an STFT and generate dynamic multi-sensor graphs using learnable dynamic graph construction, where each graph corresponds to a specific time slot. The DyGAT module further extends the application of dynamic graph structures by combining graph attention mechanisms to dynamically adjust the weights between nodes in the graph. Specifically, DyGAT establishes temporal dependencies between different time slots through DGT, effectively aggregating information across time slots through parallel processing. Additionally, DyGAT employs an adaptive learning mechanism to assign weights to the nodes in the graph, focusing on crucial spatiotemporal information within the graph and thereby enhancing the model’s ability to learn from complex sequential data. The time–frequency graph pooling layer module extracts local features from time–frequency data through convolutional operations and aggregates time–frequency information in conjunction with adjacency matrix information. This module employs adaptive weight adjustment and graph convolution mechanisms to optimize the feature representation of time–frequency graphs, improving the model’s performance in processing time–frequency data. To obtain the final fault classification result, the output module calculates the value of each category through average pooling and fully connected layers. The following sections will provide a detailed introduction to the model.

### 3.2. Learnable Dynamic Graph Construction (LDGC)

**Figure 2:**
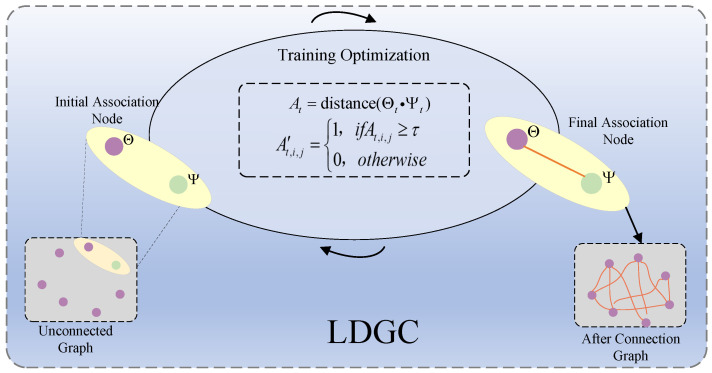
The learnable dynamic graph building module generates an adjacency matrix by calculating node similarities using randomly initialized embedding vectors and trains it for optimization.

Constructing a reasonable graph structure is crucial for modeling the relationships between samples. We propose a learnable dynamic graph construction module which generates an adjacency matrix by calculating the similarity between nodes and selecting neighbors according to a specified threshold. Unlike traditional feature-based adjacency matrix construction methods, this module encodes the adjacency matrix using learnable shallow embedding and then optimizes it through training as shown in Figure 2. Specifically, for each time slot *t*, there are two vectors Θ and Ψ of a length *d*, Here, $\Theta =[\theta_{t,1},\theta_{t,1},...,\theta_{t,d}]$ and $\Psi =[\psi_{t,1},\psi_{t,2},...,\psi_{t,d}]$ are randomly initialized learnable parameters which represent the source and target embeddings of the nodes, respectively. The initial adjacency matrix is calculated through the following formula:

(5) $$A_{t}=\mathrm{distance}\left(\Theta_{t}\cdot \Psi_{t}\right)$$

Here, distance(·) represents the similarity calculation method between the source embedding and the target embedding. The optional calculation methods include the following:

- Dot product: Calculate the similarity through the dot product;
- Cosine similarity: Calculate the similarity after normalization.

To remove self-loops from the graph, we set the diagonal values of the adjacency matrix to −∞. This ensured that there were no connections from a node to itself. Subsequently, based on a specified distance threshold τ, we selected neighbors whose similarity values were greater than or equal to τ, sparsely transforming the adjacent matrix to generate the final graph structure:

(6) $$A_{t,i,j}^{\prime }=\left\{\begin{array}{c} 1,\;\mathrm{if}\;A_{t,i,j}\geq \tau \left(\mathrm{Dot}\;\mathrm{Product}\right)\\ 1,\;\mathrm{if}\;A_{t,j,j}\leq \tau \left(\mathrm{Cos}\mathrm{ine}\;\mathrm{Similarity}\right)\\ 0,\;\mathrm{otherwise}\; \end{array}\right.$$

By employing this method, the adjacency matrix is scarified, retaining only neighbors which meet the threshold criteria and thereby significantly reducing computational costs. Furthermore, the embedding parameters Θ and Ψ are trainable, allowing the generated adjacency matrix to be continuously optimized during the training process. This enables the graph structure to better adapt to the objectives of the task.

### 3.3. DyGAT

**Figure 3:**
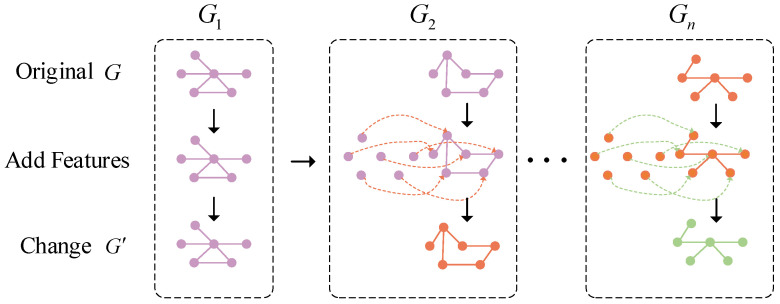
The nodes in the current graph integrate information from the corresponding nodes in the graph from the previous time slot.

This paper investigates the spatial correlations among different sensors. A transformation method was designed for dynamic graph collections to establish relationships between graphs at different time points. This strategy is based on the assumption that graph data at the current moment can be transformed by combining them with data from the previous moment. To discretize this process, a graph connection mechanism was constructed to ensure each graph aligned with the graph from the previous moment. The detailed structure of the DGT is illustrated in Figure 3.

For each time slot graph except for the first one, additional vertices were added to represent the data of corresponding vertices from the previous time slot graph. Consequently, the vertex set was modified to v(t,1), v(t,2),…, v(t,N), v(t−1,1), v(t−1,2),…, v(t−1,N). Directed edges were assigned from vertices at the previous time point to their corresponding vertices at the current time point, specifically from v(t−1,n) to v(t,n) for n=1,2,...,N. The newly generated graph set was then passed to the next processing step.

In practice, doubling the number of vertices is unnecessary in implementation. For the newly introduced directed edges, the embedded source nodes can be aggregated into the embedded target nodes, after which the additional vertices are removed.

The GAT is a GNN architecture which does not require complex matrix operations, simplifying the computational process of the model. The GAT utilizes an attention mechanism, allowing the model to assign different weights to different nodes in the graph. This enables the model to focus more on the more important or relevant parts of the graph. Inspired by the GAT and DyGIN [52], we designed a method for dynamic graph attention networks which aggregates information composed of dynamic paradigms in parallel, as shown in Equation (4). Compared with static GNNs, dynamic graph attention networks separate data from different time slots within the same dimension. In practice, the same vertex at different time slots typically aggregates information from different sets of vertices.

To adapt flexibly to the processing of dynamic graphs, we designed a group linear layer which reshapes the input tensor:

(7) $${X^{\prime }}_{group}=W_{group\;▪}\;X_{group}$$

In the group linear layer, the input features $X\in R^{B\times C_{in}\times N\times F}$ are divided into G groups, with each group having a feature dimension of F/G, resulting in $X_{group}\in R^{B\times C\times G\times N\times \frac{F}{G}}$,$W_{group}\in R^{C\times C^{\prime }}$. After reshaping, we obtain ${X^{\prime }}_{group}\in R^{B\times C^{\prime }\times N\times F}$.

DyGAT can be defined as follows:

(8) $$h_{i}^{\left(t,h\right)}=\sum_{j\in N(i)}\alpha_{ij}^{h}\cdot W_{h}{X^{\prime }}_{group}+h_{i}^{\left(t-1,h\right)}$$

(9) $$h_{i}^{\left(t\right)}={\left|\right|}_{h=1}^{H}h_{i}^{\left(t,h\right)}$$

(10) $$h_{i}^{\left(t\right)}=h_{i}^{\left(t\right)}+b$$

where $\alpha_{ij}^{h}$ represents the weight assigned by the attention mechanism to node *j* for node *i*, while $W_{h}$ denotes the linear transformation matrix of the $h_{th}$ attention head.

### 3.4. Time–Frequency Graph Pooling

**Figure 4:**
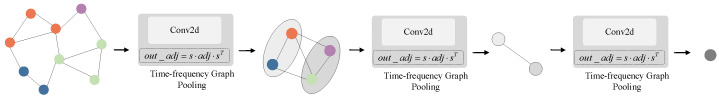
Time-frequency graph pooling employs CNNs to extract node features, introduces adaptive parameters for feature adjustment, and modifies the adjacency matrix to simulate time–frequency dependencies.

In this paper, we propose a time–frequency graph pooling method and apply it to the DyGAT-FTNet network, aiming to enhance the model’s feature learning capabilities when dealing with time–frequency graph data. The key design philosophy of the module is to extract the local features of time–frequency data through convolutional layers and to aggregate and enhance time–frequency information by combining adjacency matrix information through an adaptive weight adjustment mechanism, as shown in Figure 4.

The main components of time–frequency graph pooling include the following:

- CNN: Consider nodes as channels for extracting the features in a CNN:
  (11) $$X^{\prime }=Conv2d\left(X\right)$$
- Adaptive Parameters: In the convolutional operation, a trainable weight parameter *w* is introduced, which interacts with the convolutional kernel. By learning this weight, the representational capacity of the features can be further adjusted. This parameter is calculated through the product with the convolutional weight matrix, forming a feature adjustment mechanism which is related to the input data:
  (12) $$w\in R^{k\times 1}$$
- Adjacency Matrix Adjustment: To further enhance the model’s ability to model time–frequency dependencies, we utilize the graph’s adjacency matrix to adjust the convolutional output:
  (13) $$out\_adj=s\cdot adj\cdot s^{T}$$
  where *s* is the adjusted vector calculated through the convolutional weights and the adaptive parameters while adj represents the adjacency matrix.

## 4. Experiments

In this section, extensive experiments are conducted on four multi-sensor fault detection datasets to showcase the performance of DyGAT-FTNet. Additionally, the performance of the critical components of DyGAT-FTNet is verified through a series of ablation studies.

### 4.1. Dataset Introduction

#### 4.1.1. XJTUSuprgear Dataset

**Figure 5:**
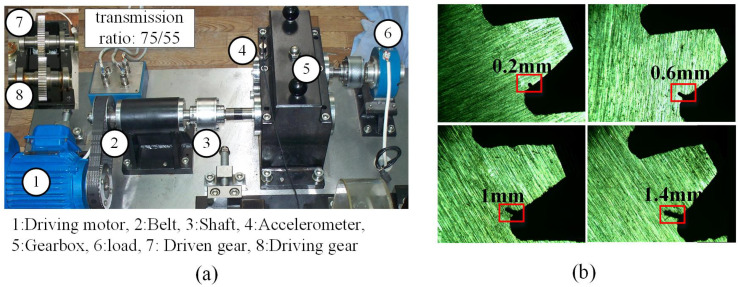
The XJTUSpurgear system simulation test bench and the types of faults. (**a**) The test rig; (**b**) Health conditions of spur-gears.

The experimental platform consists of a drive motor, a belt, a shaft, and a gearbox. The motor type is an AC variable frequency motor using single-phase AC power (220 V, 60/50 Hz), as shown in Figure 5a. Twelve one-dimensional acceleration sensors (PCB333B32) were installed on the gearbox [24]. The author has confirmed. Four root cracks of different degrees were prefabricated on the spur gear as well as the normal condition, and a total of 5 vibration signals were collected, as shown in Figure 5b. Three different speed conditions were simulated: 900 RPM, 1200 RPM, and a ramp from 0 to 1200 RPM and then back to 0. The sampling frequency during the experiment was set to 10 kHz, as shown in Table 1.

#### 4.1.2. SEU Bearing Dataset

**Figure 6:**
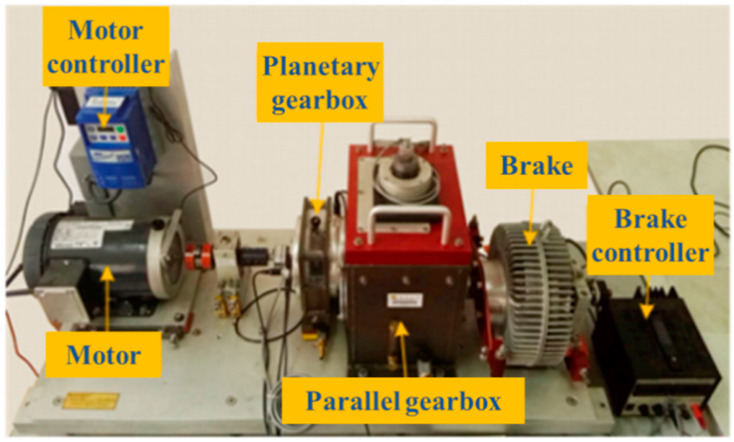
The SEU system simulation test bench.

The SEU bearing dataset was derived from the Drivetrain Dynamic Simulator (DDS), and it is shown in Figure 6. Data were collected under two distinct operating conditions: the rotating speed-system load configurations were set to 20 Hz–0 V and 30 Hz–2 V. For this experiment, the 20 Hz–0 V configuration was selected. The dataset includes four types of bearing fault conditions and one healthy condition. The specific fault states are rolling element cracks, inner race cracks, outer race cracks, and simultaneous cracks in both the inner and outer races, as detailed in Table 2.

### 4.2. Experimental Details

In the experiment, the number of dynamic graphs was set to 6, with the graph construction metric method being dot product similarity. The temporal convolution consisted of 3 layers, with kernel sizes of 11, 3, and 3. The convolutional channel sizes were 64, 128, and 256. The batch size was set to 16, and the learning rate was set to $10^{-4}$. The dataset was split into training and validation sets at a ratio of 6:4. All experiments were implemented in PyTorch 1.13 on Python 3.9.12, with a training duration of 100 epochs (computational infrastructure: Windows operating system, GPU NVIDIA RTX 3080).

### 4.3. Evaluation Indicators

In this study, DyGAT-FTNet was evaluated using two evaluation indicators—accuracy and F1 score—and a confusion matrix was plotted to compare the classification results with the actual measured values.

#### 4.3.1. ACC

Accuracy is a straightforward metric for measuring the correctness of a model’s predictions, representing the proportion of correctly classified samples out of the total number of samples. The formula for calculating the accuracy is

(14) $$\begin{array}{c} ACC=\frac{TP+TN}{TP+TN+FP+FN} \end{array}$$

where TP (true positive) represents correctly predicted positive instances, TN (true negative) represents correctly predicted negative instances, FP (false positive) represents incorrectly predicted positive instances, and FN (false negative) represents incorrectly predicted negative instances.

#### 4.3.2. F1 Score

The F1 score is the harmonic mean of the precision and recall, balancing the two when considering model performance. This is especially useful in cases of imbalanced datasets. The formula for the *F*1 score is

(15) $$\begin{array}{c} F1=2\times \frac{Precision\times Recall}{Precision+Recall} \end{array}$$

In this context, precision is the ratio of true positive predictions to the total positive predictions made by the model, calculated by

(16) $$\begin{array}{c} Precision=\frac{TP}{TP+FP} \end{array}$$

Recall, on the other hand, is the ratio of true positive predictions to all actual positive instances in the dataset and is given by

(17) $$\begin{array}{c} Recall=\frac{TP}{TP+FN} \end{array}$$

The F1 score effectively combines the precision and recall, providing a single metric which captures the performance of the model in both aspects.

### 4.4. Hyperparameter Stability

**Figure 7:**
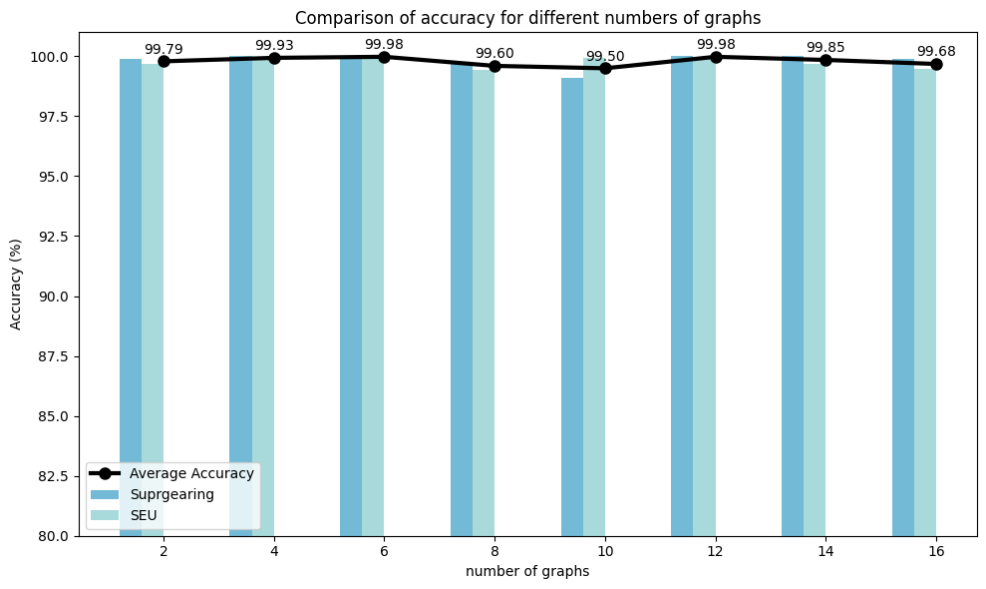
Comparison of accuracies for different numbers of graphs.

**Figure 8:**
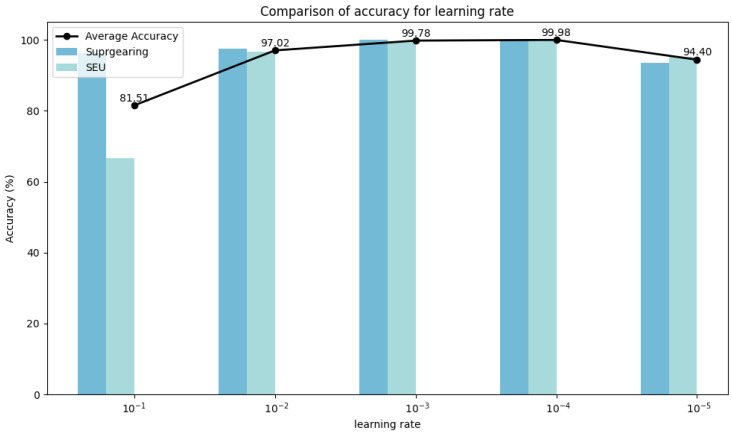
Comparison of accuracies for learning rates.

**Figure 9:**
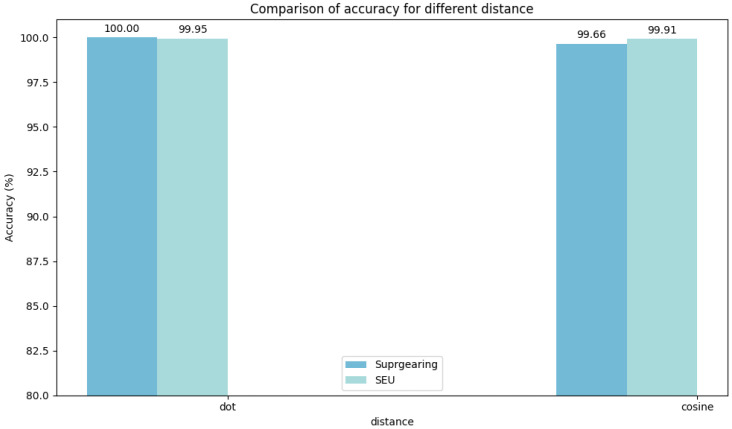
Comparison of accuracies for different distances.

**Figure 10:**
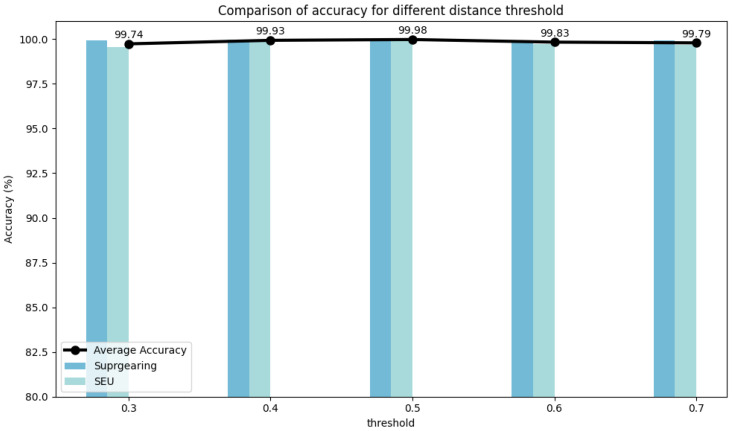
Comparison of accuracies for different distance thresholds.

**Figure 11:**
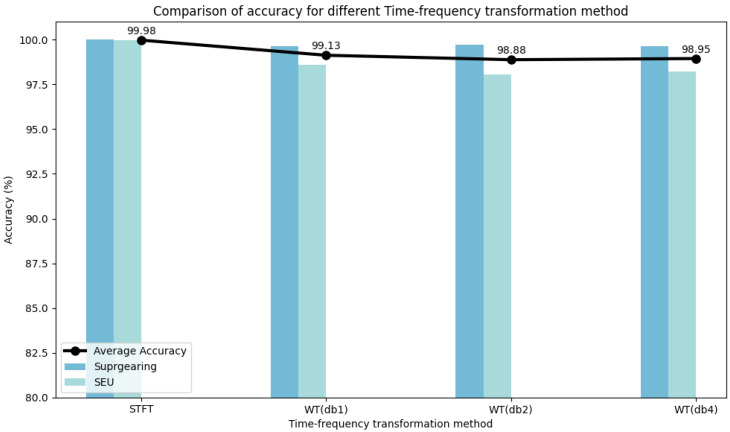
Comparison of accuracies for different time–frequency transformations.

Experiments were conducted on two selected datasets to evaluate the hyperparameter stability of DyGAT-FTNet by varying the number of dynamic graphs *G*, learning rate lr, graph construction metric distance(·), distance threshold threshold(·), and time–frequency transformation method *TF-Method*(·). It is worth noting that the number of dynamic graphs corresponds to the number of slices in the time series data. In Figure 7, Figure 8, Figure 9, Figure 10 and Figure 11, the bar charts illustrate how the classification accuracy varied with nG:{2,4,6,8,10,12,14,16}, $lr:\{10^{-1},10^{-2},10^{-3},10^{-4},10^{-5}\}$, *distance*(_▪_) : {*DotProduct*, *CosineSimilarity*}, *threshold*(_▪_) : {0.3, 0.4, 0.5, 0.6, 0.7}, and *TF* − *Method*(_▪_) : {*STFT*,*WT*(*db1*),*WT*(*db2*),*WT*(*db4*)} in all datasets, where db1, db2, and db4 represent wavelet coefficients. The black curve in the figure represents the average accuracy of DyGAT-FTNet with respect to parameter changes on the two selected datasets.

Overall, the model performance remained consistently high. When datasets with significant length differences were divided into equal time periods, the average accuracy fluctuated slightly as the nG value increased. The changes in the two graph construction metrics had a minimal impact on the model accuracy. However, the dot product method demonstrated higher accuracy and versatility. As the distance threshold varied, the average accuracy fluctuated slightly, with the highest accuracy observed at a threshold of 0.5. Similarly, in the time–frequency transformation, the model maintained high accuracy across different methods, with the STFT method outperforming the wavelet transform method in terms of both accuracy and versatility. Overall, the implicit dependencies extracted by DyGAT-FTNet proved highly effective for multi-sensor fault detection and classification tasks. Moreover, the results indicate that optimal lr values contribute to achieving better classification accuracy.

Additionally, Table 3 compares the model parameter counts and training times between the two datasets. The table presents the number of parameters and the training times for the XJTUSuprgear and SEU datasets. The results indicate that although the XJTUSuprgear dataset requires more parameters and training time due to its larger size, the model performed efficiently on both datasets. The results also highlight the significant computational costs associated with processing more sensors or longer time series data.

### 4.5. Ablation Study

To verify the effectiveness of the key components of DyGAT-FTNet, ablation studies were performed on two datasets. DyGAT-FTNet was evaluated with different components as follows:

- **No dynamic graph construction:** DyGAT-FTNet did not include the dynamic graph construction module, and KNN graph construction was used as one of the inputs for the graph neural network.
- **No dynamic graph attention mechanism**: The graph attention mechanism was directly used without dynamic adjustment.
- **No time–frequency graph pooling layer**: The time–frequency graph pooling layer was bypassed, and the outputs of the graph neural network were directly connected to the output module.

As shown in Table 4, LGDC achieved better model performance than static graphs as it facilitated feature flow between independent yet interrelated sensors. The dynamic graph attention network extracted richer feature information from dynamic graphs compared with traditional graph attention networks. Time–frequency graph pooling demonstrated its effectiveness by aggregating central data in a hierarchical manner, significantly enhancing GNN performance. In summary, the ablation experiment results confirm the effectiveness of the proposed DyGAT-FTNet components in multi-sensor fault diagnosis tasks.

### 4.6. Comparative Experiments

To evaluate the performance of DyGAT-FTNet, the proposed model was compared with baseline models on the selected datasets, with the main results presented in Table 5. The specific accuracy of the baseline models is referenced from the original paper. The best results are highlighted in bold. The baseline models selected for comparison include: MLP [53], GCN [25], ChebyNet [54], GraphSage [55], A-TSGNN [28], GATU-Net [56], Todynet [52] and ETMD [57].

Table 5 presents the comparative accuracies of different models on the two datasets. The performance of each model varied. MLP has a limited ability to handle complex time–frequency data, resulting in relatively low accuracy and F1 scores. Although the GCN is effective for graph-structured data, its performance on dynamic time–frequency tasks is limited due to its inability to effectively model temporal dependencies. GraphSage outperformed ChebyNet by leveraging a neighborhood aggregation strategy, achieving higher accuracy and F1 scores. However, its reliance on the fixed structure of the graph limits its flexibility for dynamic datasets. A-TSGNN performed well on the SEU dataset, but it requires many computing resources. GATU-Net performed quite well, and weighted shrinkage loss (WSKloss) was able to adaptively adjust the focus on difficult-to-classify samples. Todynet achieved near-perfect performance across both datasets. ETMD is a model based on the Transformer architecture, but the effect was not satisfactory in this experiment. However, the proposed DyGAT-FTNet outperformed all other models on both datasets, achieving an accuracy of 1.00 on the XJTUSuprgear dataset and minimal error on the SEU dataset. These results demonstrate the effectiveness of DyGAT-FTNet in multi-sensor fault detection tasks and highlight its superior capability to process complex time–frequency data and uncover hidden dependencies among multiple sensors.

**Figure 12:**
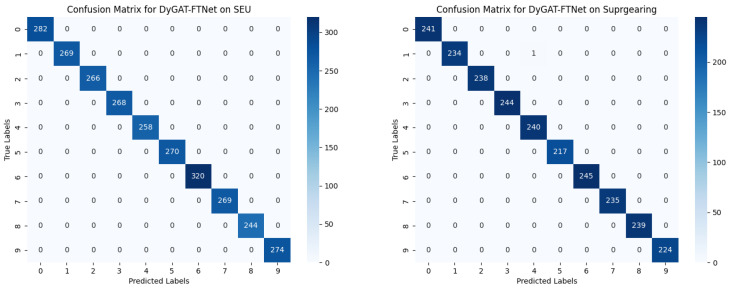
(**a**) The confusion matrix for the SEU dataset. (**b**) The confusion matrix for XJTUSpurgearing dataset. DyGAT-FTNet performed well on both datasets.

To examine the separability of the proposed model for normal and fault data, a confusion matrix was used to display the detailed diagnostic results of the two experimental datasets, as shown in Figure 12. In the confusion matrix, the numbers on the main diagonal represent correct classifications, while the off-diagonal numbers indicate misclassifications. As depicted in Figure 12, DyGAT-FTNet effectively distinguished fault types in the two datasets and accurately differentiated between speed types.

**Figure 13:**
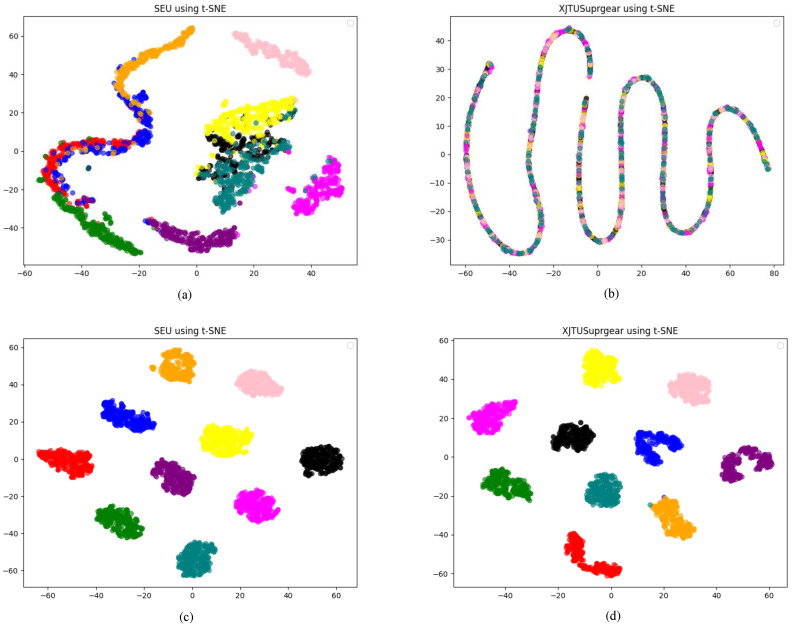
(**a**) The original feature map of the SEU dataset, (**b**) The feature map of the SEU dataset after extraction in DyGAT-FTNet, (**c**) The original feature map of the XJTUSuprgear dataset. (**d**) The feature map of the XJTUSuprgear dataset after extraction in DyGAT-FTNet. This figure illustrates the transformation and enhancement of feature maps through the DyGAT-FTNet extraction process, highlighting the differences between the original and processed feature maps for both the SEU and XJTUSuprgear datasets. The comparison demonstrates the effectiveness of DyGAT-FTNet in refining and extracting more meaningful features from the original data.

Additionally, t-distributed stochastic neighbor embedding (t-SNE) was employed to visualize the features learned by DyGAT-FTNet during training. Each row in Figure 13 represents a different dataset, and each column visualizes the original data alongside the embeddings learned by DyGAT-FTNet. The visualization reveals that samples from the same class clustered closely, demonstrating DyGAT-FTNet’s ability to classify data with high accuracy.

### 4.7. Noise Resistance

**Figure 14:**
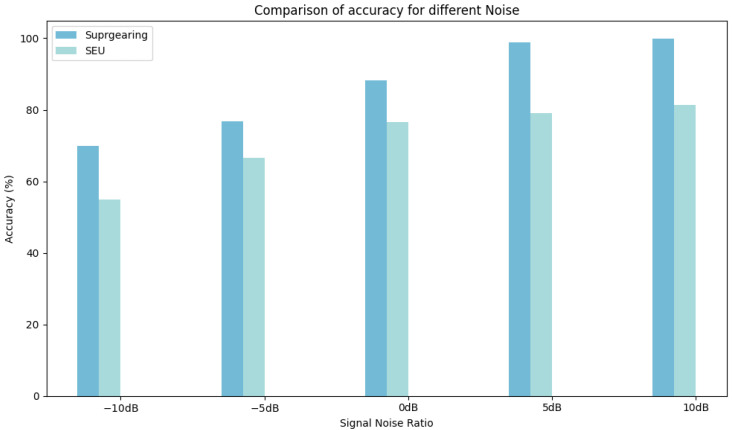
Comparison of accuracies for different signal-to-noise ratios.

To evaluate the robustness of DyGAT-FTNet against noise, we conducted experiments on two datasets. In this experiment, Gaussian white noise was added to the original vibration signal at varying signal-to-noise ratios (SNRs), ranging from −10 dB to 10 dB. The experimental results are presented in Figure 14.

It was observed that the performance of DyGAT-FTNet decreased as the noise increased and improved as the noise decreased. The model performed more stably at higher SNRs, indicating its noise resistance and ability to maintain high accuracy in noisy environments. However, at lower SNRs (e.g., −10 dB), the model performed poorly, suggesting that DyGAT-FTNet’s noise resistance requires improvement under extremely low noise conditions.Therefore, although the model exhibited noise resistance, further optimization is needed for extremely low noise conditions in the future.

## 5. Conclusions

This paper introduced a multi-sensor fault detection method, DyGAT-FTNet, and proposed an effective strategy to reveal the hidden dependencies between multi-sensor data and time–frequency dynamic features at different time periods. DyGAT was used to extract time–frequency dynamic features. In addition, an innovative time-frequency graph pooling method was designed to effectively overcome the flatness problem faced by traditional graph neural networks when processing time–frequency data. Compared with other methods, our method performed well on two datasets.

However, DyGAT-FTNet still has some limitations which require further exploration. First, the model has poor noise immunity, which affects its performance in noisy environments. Second, the high computational complexity of DyGAT-FTNet may hinder its deployment in real-time applications or on resource-constrained devices. Lastly, the model’s generalization capability across different datasets and sensor configurations remains unclear, as DyGAT-FTNet has not been tested on datasets from other industries. These limitations highlight the need for continued research to address these challenges and enhance the model’s applicability.

For future research directions, we propose three main focuses:

1. Improving model noise resistance: One of the primary challenges for DyGAT-FTNet is its limited noise resistance, which impacts its performance in noisy environments. Future work should explore methods to enhance the model’s robustness to noise, such as incorporating noise-resistant feature extraction techniques, enhancing the model’s ability to filter out irrelevant noise, or applying advanced noise reduction algorithms. Additionally, hybrid models combining DyGAT-FTNet with noise-robust techniques such as denoising autoencoders or attention mechanisms could further improve the model’s performance in real-world applications where noise is inevitable.

2. Sensor selection and optimization: In multi-sensor scenarios, the presence of redundant or inefficient sensors may adversely affect the performance of the model and increase the computational cost. Sensor selection and optimization research, including a preliminary analysis and heuristic approach, is critical to improving the efficiency and scalability of DyGAT-FTNet.

3. Model generalization: Future research should focus on the generalization capability of DyGAT-FTNet across different datasets and sensor configurations. While the model showed good performance on the XJTUSuprgear and SEU datasets, it remains to be seen how well it can be applied to datasets from other industries or with different sensor set-ups. Conducting experimental studies across diverse industries or sensor configurations to validate the model’s applicability and robustness will help improve its potential for widespread adoption.

In summary, while DyGAT-FTNet demonstrates strong performance in multi-sensor fault detection tasks, addressing the limitations related to computational complexity, noise resistance, and generalization capability, as well as exploring the proposed future research directions, will further enhance its practicality and adaptability in real-world applications.

## Figures and Tables

**Table 1:** XJTUSuprgear dataset.

Label	Fault Description	Speed
0	Normal	900 RPM
1	Gear tooth root with 0.2 mm cracks	900 RPM
2	Gear tooth root with 0.6 mm cracks	900 RPM
3	Gear tooth root with 1.0 mm cracks	900 RPM
4	Gear tooth root with 1.4 mm cracks	900 RPM
5	Normal	1200 RPM
6	Gear tooth root with 0.2 mm cracks	900 RPM
7	Gear tooth root with 0.6 mm cracks	900 RPM
8	Gear tooth root with 1.0 mm cracks	900 RPM
9	Gear tooth root with 1.4 mm cracks	900 RPM

**Table 2:** SEU dataset.

Label	Fault Description	Speed
0	Normal	20 Hz
1	Cracks in rolling element	20 Hz
2	Cracks in the inner ring	20 Hz
3	Cracks in the outer ring	20 Hz
4	Cracks appear in both the inner and outer rings	20 Hz
5	Normal	30 Hz
6	Cracks in rolling element	30 Hz
7	Cracks in the inner ring	30 Hz
8	Cracks in the outer ring	30 Hz
9	Cracks appear in both the inner and outer rings	30 Hz

**Table 3:** Comparison of model parameters and training times between XJTUSuprgear and SEU datasets.

Dataset	Number of Sensors	Number of Parameters	Training Time (in Seconds)
XJTUSuprgear	12	444,086	7556.498
SEU	8	442,684	5114.916

**Table 4:** Ablation Study results.

Model	XJTUSuprgear		SEU	
	ACC	F1	ACC	F1
No dynamic graph construction	0.9659	0.9634	0.9457	0.9457
No dynamic graph attention mechanism	0.9735	0.9734	0.9877	0.9877
No time–frequency graph pooling layer	0.9775	0.9776	0.9296	0.9293
DyGAT-FTNet	1.0000	1.0000	0.9995	0.9996

**Table 5:** Comparative Experimental Results.

Model	XJTUSuprgear		SEU	
	ACC	F1	ACC	F1
MLP	0.3084	0.2728	0.7800	0.7523
GCN	0.1207	0.1019	0.6300	0.5673
ChebyNet	0.3336	0.2788	0.8500	0.8215
GraphSage	0.8746	0.6565	0.8500	0.8458
A-TSGNN	-	-	0.9927	0.9920
Todynet	0.9965	0.9966	0.9929	0.9929
GATU-Net	0.9998	0.9998	-	-
ETMD	0.9958	0.9814	0.9440	0.9278
DyGAT-FTNet	1.0000	1.0000	0.9995	0.9996

## Data Availability

The SEU dataset used in the experiments of this paper is available at https://github.com/cathysiyu/Mechanical-datasets, accessed on 26 January 2025. The XJTUSuprgear dataset used in the experiments of this paper is available at https://drive.google.com/drive/folders/1ejGZu9oeL1D9nKN07Q7z72O8eFrWQTay, accessed on 26 January 2025.
